# Prenatal influenza exposure and cardiovascular events in adulthood

**DOI:** 10.1111/irv.12202

**Published:** 2013-11-07

**Authors:** Noelle M Cocoros, Timothy L Lash, Al Ozonoff, Mette Nørgaard, Alfred DeMaria, Viggo Andreasen, Henrik Toft Sørensen

**Affiliations:** a Massachusetts Department of Public Health, Bureau of Infectious DiseaseJamaica Plain, MA, USA; b Department of Epidemiology, Rollins School of Public Health, Emory UniversityAtlanta, GA, USA; c Center for Patient Safety and Quality Research, Program for Patient Safety and Quality, Boston Children's HospitalBoston, MA, USA; d Department of Pediatrics, Harvard Medical SchoolBoston, MA, USA; e Department of Clinical Epidemiology, Aarhus University HospitalAarhus, Denmark; f Department of Science, Systems, and Models, Roskilde UniversityRoskilde, Denmark

**Keywords:** Acute myocardial infarction, influenza, pandemic, stroke

## Abstract

**Objectives:**

This study examined the association between prenatal exposure to pandemic influenza and cardiovascular events in adulthood.

**Design:**

Using Danish surveillance data to identify months when influenza activity was highest during three previous pandemics (1918, 1957, and 1968), persons were defined as exposed/unexposed based on whether they were *in utero* during peak months of one of the pandemics. Episodes of acute myocardial infarction (MI) and stroke were identified in the Danish National Registry of Patients covering all Danish hospitals since 1977.

**Setting/Sample:**

Information from Danish national registries on all persons with a Civil Personal Registry number and birthdates in 1915 through 1922, 1954 through 1960, and 1966 through 1972 was collected.

**Main outcome measures:**

Crude incidence rate ratios (IRRs) were calculated per pandemic. Generalized linear models were fit to estimate IRRs adjusted for sex.

**Results:**

For acute MI, sex-adjusted IRRs for persons *in uter*o during peaks of the 1918, 1957, and 1968 pandemics, compared with those born afterward, were 1·02 (95% confidence interval (CI): 0·99, 1·05), 0·96 (95% CI: 0·87, 1·05), and 1·18 (95% CI: 0·96, 1·45), respectively. For stroke, the corresponding IRRs were 0·99 (95% CI: 0·97, 1·02), 0·99 (95% CI: 0·92, 1·05), and 0·85 (95% CI: 0·77, 0·94), respectively.

**Conclusions:**

There was generally no evidence of an association between prenatal influenza exposure and acute MI or stroke in adulthood. However, survivor bias and left truncation of outcomes for the 1918 pandemic are possible, and the current young ages of persons included in the analyses for the 1957 and 1968 pandemics may warrant later re-evaluation.

## Introduction

Influenza can be especially serious for pregnant women.^1^,^2^ During the 2009 H1N1 pandemic, pregnant women experienced disproportionate hospitalization and mortality,^3^–^5^ similar to reports from the 1918 and 1957 influenza pandemics.^6^–^8^ Studies also have demonstrated an association between influenza virus infection and spontaneous abortion, preterm birth, stillbirth, and low birth weight.^5^,^9^–^14^ Furthermore, influenza can be serious for neonates and infants.^15^,^16^ While the acute effects of influenza on pregnant women and their children are well established, a growing literature is examining possible long-term consequences for prenatally exposed offspring.

The impact of prenatal exposure to maternal influenza virus infection has been evaluated using a variety of approaches. Numerous studies have examined the relationship between *in utero* exposure to the 1957 influenza pandemic and development of schizophrenia, with conflicting results.^17^–^22^ Several researchers have examined birth cohort effects associated with the 1918 pandemic. A birth cohort study encompassing 24 countries found no evidence of increased late-life mortality among those exposed prenatally or shortly after birth to the 1918 pandemic.^23^ A study examining birth cohort effects among 60- to 82-year-olds born in the USA between 1915 and 1923 found that persons born in 1919, who would have been *in utero* during the 1918 pandemic, were more likely to report negative health outcomes (*e.g*., fair-to-poor health; trouble hearing) compared with those born in 1915–1918 and 1920–1923.^24^ A time-series analysis of prenatal exposure to the 1918 pandemic showed an increased rate of self-reported prevalent cardiovascular disease among American adults born in 1919.^25^

This study was designed to examine whether having been *in utero* during an influenza pandemic, a proxy for prenatal influenza exposure, is associated with an increased risk of physician-diagnosed acute myocardial infarction (MI) or stroke. We used Danish surveillance data to identify months when influenza activity was highest during three previous pandemics (1918, 1957, and 1968) and defined persons as exposed or unexposed based on birth month and year.

## Methods

### Study population and data collection

We collected information from Danish national registries on all persons with a Civil Personal Registry (CPR) number and birthdates in 1915 through 1922, 1954 through 1960, and 1966 through 1972. The CPR number, a unique personal identifier assigned to all Danish residents at birth or upon immigration, was introduced in April 1968,^26^ allowing linkage between administrative, medical, and other registries. Data available as of January 1, 2010 were included in the analytic dataset. From the Civil Registration System, we obtained information on date of birth, vital status (alive, died, emigrated, not residing in Denmark, or lost to follow-up), dates of changes in vital status, and sex.

### Outcome variables

Outcomes of interest were a first-time diagnosis of acute myocardial infarction (MI), and cerebrovascular events [stroke or transient ischemic attack (TIA)] recorded for a hospitalization or a hospital outpatient visit. Stroke and TIA were combined in our analyses. The Danish National Registry of Patients (DNRP) contains information on all hospitalizations since 1977 and on outpatient visits at hospital clinics and emergency rooms since 1995. Since 1994, diagnoses have been classified according to the *International Classification of Diseases*, 10th revision (ICD-10), replacing *International Classification of Diseases,* 8th revision (ICD-8) codes. We used the following diagnostic codes to identify the first diagnosis of acute MI events: ICD-8 code 410 and ICD-10 codes I21·0–I21·9. For incident stroke and TIA, we used ICD-8 codes 430–434 and 436, and ICD-10 codes I60–I69 and G45.

### Exposure variables

**Figure 1:**
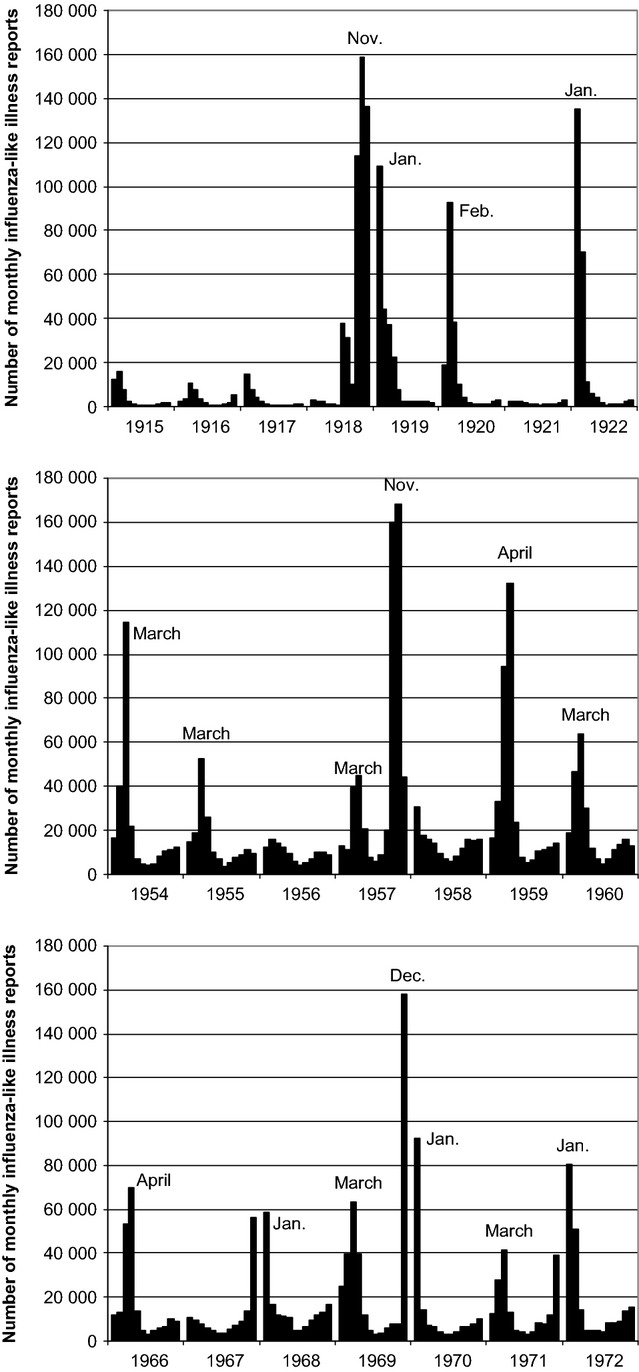
Physician-reported cases of influenza-like illness, by month, 1915–1922, 1954–1960, and 1966–1972, Denmark.

Exposed and unexposed persons were identified based on their month and year of birth. We obtained historical monthly surveillance data on physician-reported cases of influenza-like illness (ILI) in Denmark for the years 1915 through 1922, 1954 through 1960, and 1966 through 1972^27^ (Figure 1). The original data contain ILI counts by three broad geographic regions (Copenhagen, ‘other towns,’ and ‘rural districts’), and for the 1915–1922 and 1954–1960 data, by age group. We visually examined the monthly ILI data and identified peaks of activity during each pandemic. Persons *in utero* for at least one of the months of peak activity were defined as exposed. Persons *in utero* only during the months flanking the peak months, when ILI activity was lower (‘buffer’ months), were excluded. Only persons whose gestation period did not include either pandemic peak or buffer months, and who were born *after* the pandemic peaks, were classified as unexposed. (There was ILI reported during some of the unexposed periods for this study, particularly in March and April 1959. However, the unexposed periods generally had substantially less ILI reported, and the ILI activity that was reported outside of the peak pandemic months was more common among older adults than children and adolescents.) Those born before one of the pandemics were excluded from the primary analyses because they could have been infected with influenza as infants or young children. We assumed that all persons were full term (i.e., *in utero* for 9 months).

The number of cases that occurred during the study period determined the sample size.

### Person-time and age restrictions

Because the DNRP does not include information on diagnoses before 1977, we restricted the age range during which events could be captured. Using the 1918 pandemic as an example, those born in 1915 (the oldest persons experiencing this pandemic) would have been 62 when the DNRP was established. Thus, we limited members of the 1915 through 1922 cohorts to persons surviving to age 62. Those born in 1922 were 62 in 1984 and could be followed until December 31, 2009. Accordingly, the maximum amount of time a person in the 1922 birth cohort could contribute to follow-up was 26 years (age 62–87). We evaluated the 1957 pandemic for events occurring among cohort members between the ages of 23 and 49, and the 1968 pandemic for events occurring among cohort members between the ages of 11 and 37. Members of the 1957 and 1968 cohorts had a maximum of 27 person-years at risk. All persons included in the study were followed from the age when they began to contribute follow-up time until death, a study outcome, emigration, or January 1, 2010, whichever occurred first.

### Statistical analysis

In similar studies, seasonality has been included to account for seasonal variation in outcomes.^22^,^25^ We assessed the presence of seasonality in the outcomes of interest among unexposed persons born after pandemic peaks by plotting outcome rates by birth month and quarter. For the 1957 and 1968 analyses, we also estimated the peak-to-low ratios and corresponding 95% confidence intervals (CI) by birth month.^28^ We could not fully assess seasonality for the 1918 analyses because we did not have a full year of data among those born after the pandemic peaks.

We computed crude incidence rate ratios (IRRs), total and stratified by sex, with 95% CI, for each pandemic. To obtain IRRs adjusted for sex, we fit generalized linear models with a Poisson distribution using SAS version 9.2 (SAS Institute, Inc., Cary, NC, USA). For each model, we created two separate two-level terms (male exposed and unexposed; female exposed and unexposed). The dependent variable was the number of outcomes, predicted by exposure and sex, with an offset term (log of person-years).

To evaluate potential consequences of misclassification of ILI exposure *in utero*, we performed a bias analysis that assumed non-differential, independent misclassification. We used ranges of sensitivity (e.g., for the 1918 cohort, these were 0·8, 0·85, 0·9, and 0·95) and specificity (e.g., for the 1918 cohort, these were 0·3, 0·35, 0·4, and 0·45) of misclassification of ILI exposure *in utero* for each of the three pandemic periods and for both outcomes. These ranges were based on published information on rates of influenza infection^29^–^33^ and so were different for each cohort. The range of sensitivities for the 1918 cohort implies that, among those truly exposed to ILI *in utero*, we correctly classified between 80% and 95%. The range of specificities for the 1918 cohort implies that, among those truly unexposed to ILI *in utero*, we correctly classified between 30% and 45%. For each of the pairwise combinations of sensitivity and specificity, we calculated an odds ratio ‘corrected’ for these misclassification rates using standard formulas for correction of misclassified binary variables.^34^ We applied this method of evaluating the bias due to misclassification of *in utero* exposure to ILI for both the MI and stroke outcomes in all three cohorts. For these analyses, we used the limited age ranges and the maximum amount of person-time at risk.

This study was approved by the Danish Data Protection Agency.

## Results

We identified 1 530 189 persons who lived in Denmark as of April 2, 1968, when the Civil Registration System was established. Table 1 shows the number of persons and amount of person-time contributed in the exposed and unexposed groups for each pandemic period. The average number of person-years contributed per person was similar for exposed and unexposed persons within each period.

**Table 1:** Characteristics of the study population

	Total (n) before restrictions	Exposed				Unexposed			
			
1918	Born 1915–1922: 470 390*	Male	Female	Total	Average person-years of follow-up	Male	Female	Total	Average person-years of follow-up
Acute MI	158 885	54 315	59 101	113 416	16·49	21 505	23 934	45 439	16·53
Stroke	160 216	55 161	59 184	114 345	16·27	21 930	23 941	45 871	16·28

	Total (n) before restrictions	Exposed				Unexposed			
			
1957	Born 1954–1960: 517 719*	Male	Female	Total	Average person-years of follow-up	Male	Female	Total	Average person-years of follow-up
Acute MI	222 718	33 880	32 173	66 053	25·25	79 897	76 768	156 665	25·22
Stroke	222 671	33 876	32 164	66 040	25·20	79 882	76 749	156 631	25·17

	Total (n) before restrictions	Exposed				Unexposed			
			
1968	Born 1966–1972: 542 080*	Male	Female	Total	Average person-years of follow-up	Male	Female	Total	Average person-years of follow-up
Acute MI	283 749	66 407	62 767	129 174	25·48	79 238	75 337	154 575	25·45
Stroke	283 727	66 401	62 764	129 165	25·46	79 233	75 329	154 562	25·42

* All persons living in Denmark, April 1968 (start of Civil Registration System); 1918: events at ages 62–87 (maximum of 26 years at risk); 1957: events at ages 23–49 (maximum of 27 years at risk); 1968: events at ages 11–37 (maximum of 27 years at risk).

Figure 1 shows the data used to identify exposure groups. The date ranges used to define exposed and unexposed groups for each pandemic are provided in Table 2.

**Table 2:** Birth date ranges used to define exposure, by influenza pandemic

Pandemic	Exposed	Unexposed
1918	October 1918–November 1920	March 1921–December 1921
1957	October 1957–August 1958	November 1958–December 1960
1968	January 1969–October 1970	December 1970–December 1972

Among persons born after the peaks of the pandemics, rates of MI and stroke were plotted by birth month and by birth-month quarter to assess presence of seasonality for the outcomes under study. Seasonality was not visually evident in any of the cohorts born after the three pandemics. For birth dates from January 1959 through December 1960, the peak-to-low ratios for acute MI and stroke were 1·10 (95% CI 1·00, 1·26) and 1·11 (95% CI 1·01, 1·23), respectively. For birth dates from January 1971 through December 1972, the peak-to-low ratios for acute MI and stroke were 1·04 (95% CI 1·00, 1·52) and 1·10 (95% CI 1·00, 1·31), respectively. We did not include adjustment for seasonality in our models. For all three pandemics, the crude, sex-stratified, and sex-adjusted IRRs and 95% CIs were generally very similar for acute MI and stroke (Table 3). There was no apparent difference in the rate of acute MI for those exposed prenatally to influenza compared with those unexposed during the 1918 and 1957 pandemics. The sex-adjusted rate ratios for 1918 and 1957 were 1·02 (95% CI: 0·99, 1·05) and 0·96 (95% CI: 0·87, 1·05), respectively. For 1968, the adjusted IRR was 1·18 (95% CI: 0·96, 1·45).

**Table 3:** Crude and adjusted incidence rate ratios for (A) prenatal influenza exposure and acute myocardial infarction and (B) prenatal influenza exposure and stroke

	Crude IRR (95% CI)			Sex-adjusted IRR (95% CI)	Cases (n)	
			
	Male	Female	Total		Exposed	Unexposed
(A)						
1918	1·04 (1·00, 1·08)	0·98 (0·93, 1·02)	1·02 (0·99, 1·05)	1·02 (0·99, 1·05)	15 940	6288
1957	0·94 (0·89, 1·05)	1·02 (0·85, 1·22)	0·96 (0·87, 1·05)	0·96 (0·87, 1·05)	597	1475
1968	1·24 (0·97, 1·59)	1·05 (0·72, 1·54)	1·18 (0·96, 1·45)	1·18 (0·96, 1·45)	180	182
(B)						
1918	0·99 (0·96, 1·03)	0·99 (0·96, 1·03)	0·99 (0·97, 1·02)	0·99 (0·97, 1·02)	22 759	9211
1957	1·05 (0·96, 1·15)	0·92 (0·83, 1·01)	0·99 (0·92, 1·05)	0·99 (0·92, 1·05)	1223	2940
1968	0·86 (0·75, 0·99)	0·84 (0·73, 0·96)	0·85 (0·77, 0·94)	0·85 (0·77, 0·94)	709	997

IRRs, incidence rate ratios.

Restricted age ranges and maximum person-time at risk were applied, as described in the Methods section.

There was no indication of increased cerebrovascular stroke risk for persons prenatally exposed to the 1918 and 1957 influenza pandemics compared with the unexposed; sex-adjusted IRRs were 0·99 (95% CI: 0·97, 1·02) and 0·99 (95% CI: 0·92, 1·05), respectively. For 1968, the adjusted IRR was 0·85 (95% CI: 0·77, 0·94).

The results of the analyses to evaluate the potential bias due to misclassification of exposure to ILI *in utero* generally yielded ‘corrected’ odds ratios that were very similar to the observed rate ratios for all pairwise combinations of sensitivity and specificity, for both MI and stroke outcomes, and in all three cohorts (Table 4). This result indicates that, assuming an accurate bias model, non-differential and independent exposure misclassification is unlikely to explain the near null associations that we have reported. At the low end of the range for specificity (0·6) for the 1968 cohort, corrected odds ratios were further from the null. This is likely explained by the sparse number of outcomes, which can unduly influence this type of bias analysis.^35^

**Table 4:** Bias analysis with corrected odds ratios for non-differential, independent exposure misclassification for the (a) 1918 pandemic and acute myocardial infarction, (b) 1918 pandemic and stroke, (c) 1957 pandemic and acute myocardial infarction, (d) 1957 pandemic and stroke, (e) 1968 pandemic and acute myocardial infarction, and (f) 1968 pandemic and stroke

Specificity	Sensitivity			

	0·80	0·85	0·90	0·95
(A)				
0·30	1·33	1·31	1·30	1·30
0·35	1·11	1·09	1·08	1·08
0·40	1·08	1·06	1·05	1·05
0·45	1·07	1·05	1·04	1·04
(B)				
0·30	0·82	0·83	0·83	0·83
0·35	0·94	0·95	0·95	0·96
0·40	0·96	0·96	0·97	0·97
0·45	0·96	0·97	0·97	0·98

	Sensitivity				
	
	0·55	0·65	0·75	0·85	0·95
(C)					
0·75	0·80	0·80	0·80	0·81	0·81
0·80	0·88	0·89	0·89	0·90	0·90
0·85	0·91	0·92	0·93	0·93	0·93
(D)					
0·75	0·93	0·93	0·93	0·93	0·94
0·80	0·96	0·96	0·96	0·97	0·97
0·85	0·97	0·97	0·97	0·98	0·98

	Senstivity			
	
	0·65	0·75	0·85	0·90
(E)				
0·60	2·25	2·06	1·97	1·95
0·65	1·79	1·63	1·57	1·55
0·70	1·62	1·48	1·42	1·40
0·75	1·54	1·41	1·35	1·33
(F)				
0·60	0·23	0·25	0·26	0·26
0·65	0·52	0·55	0·57	0·57
0·70	0·62	0·66	0·67	0·68
0·75	0·67	0·71	0·73	0·74

(A) Uncorrected: OR: 1·02; IRR: 1·02; sex-adjusted IRR: 1·02 (95% CI: 0·99, 1·05).

(B) Uncorrected: OR: 0·99; IRR: 0·99; sex-adjusted IRR: 0·99 (95% CI: 0·97, 1·02).

(C) Uncorrected: OR: 0·96; IRR: 0·96; sex-adjusted IRR: 0·96 (95% CI: 0·87, 1·05).

(D) Uncorrected: OR: 0·99; IRR: 0·99; sex-adjusted IRR: 0·99 (95% CI: 0·92, 1·05).

(E) Uncorrected: OR: 1·18; IRR: 1·18; sex-adjusted IRR: 1·18 (95% CI: 0·96, 1·45).

(F) Uncorrected: OR: 0·85; IRR: 0·85; sex-adjusted IRR: 0·85 (95% CI: 0·77, 0·94).

## Discussion

In this study, we found generally no evidence of an association between prenatal influenza exposure and acute MI or stroke in later life. This was true across the pandemics, although the results for the 1968 pandemic suggested a slightly protective effect for stroke.

A major advantage of our study was the availability of person-time for every Danish resident surviving to approximately 1977, starting from the birth years of interest. We based exposure definitions on surveillance data from a national source and the periods of high activity identified in this dataset correspond well with other published reports on pandemics in Denmark and other parts of Europe.^9^,^31^,^36^

Being *in utero* during a peak influenza period is a proxy for actual prenatal exposure. Thus, there is certain to be exposure misclassification for both the exposed and unexposed groups. However, we improved the exposure classification by excluding persons who would have been *in utero* during ‘buffer’ months, when ILI was at varying levels in the months immediately before and after the peaks. The exposure misclassification is likely non-differential and independent, and the results of our bias analysis were generally consistent with our observed data.

Use of the DNRP to identify the outcomes of interest has both strengths and limitations. While there is clear benefit to using physician-diagnosed events, misclassification (albeit likely non-differential) is a concern, particularly for stroke diagnoses. Two research groups have demonstrated that the quality of the stroke and TIA data in the DNRP is low for some diagnostic subgroups and the data should be used with caution.^37^,^38^

The potential for healthy survivor bias, particularly in the 1918 pandemic analysis, must be considered. In Denmark, there was a decrease in the birth rate in 1919, followed by an increase in 1920. This trend has been noted in other European countries that, like Denmark, were not heavily involved in World War I.^39^ In 1919, Harris reported an increased risk of miscarriage or premature birth in pregnant women who had influenza complicated by pneumonia during the 1918 pandemic.^7^ The results of a recent study provide compelling evidence that the birth rate decline in Denmark in 1919 was caused by miscarriages among women with influenza during their first trimester.^9^ If many of the fetuses exposed to the 1918 pandemic were miscarried, and there is indeed an association between prenatal influenza exposure and the outcomes of interest, this effect would not be apparent in our analyses. In addition, any perinatal mortality occurring as a direct result of prenatal or perinatal infection, or as an indirect result of prenatal infection via premature birth, could also contribute to a healthy survivor effect. Unfortunately, perinatal mortality data are not readily available. Birth rate declines were not apparent around the time of the 1957 and 1968 pandemics, so we do not believe survivor bias is present in analyses for those pandemics.

Another important factor related to the 1918 pandemic analysis is left truncation of the outcomes. The DNRP was established in 1977, so we lacked information on MI or stroke events occurring before that year. The 1918 pandemic-related cohorts were in the prime age for cardiovascular disease during the post-World War II period when the developed world experienced an epidemic of cardiovascular disease. Because this occurred before 1977, these outcomes could not be included in the present study. Danish cause-of-death data are available in hard-copy form from 1943 on and could provide the basis for an important follow-up to the analysis reported here. At the same time, persons included in the 1957 and 1968 pandemic analyses are still relatively young for the outcomes of interest in this study. However, while it is true that the risk for some of these outcomes is low in the ages attained by the exposed cohorts, it should also be noted that low-risk populations have been endorsed for studies of weak associations.^40^

Data are unavailable on variables such as socioeconomic status and other variables of interest such as birth weight (the Danish Medical Birth Registry began in 1973). While such factors may be important for the question at hand, and may be effect modifiers, we are not very concerned with residual confounding in this study because there do not appear to be factors that would be associated with both birth year and the outcomes of interest. Finally, any changes to the health system or to health habits over time would have affected the exposed and unexposed nearly uniformly. The unexposed cohorts were born within a couple years of the exposed cohorts, so these social and healthcare changes would have impacted both nearly identically.

In the 1980s and 1990s, Barker and colleagues articulated what has come to be known as the fetal origins hypothesis.^41^ The hypothesis states that adverse prenatal conditions can ‘program’ the fetus and cause physical and metabolic changes during key developmental stages that result in increased risk of certain conditions such as hypertension and diabetes. There is accumulating evidence regarding environmental exposures and the development of cardiovascular disease, including prenatal exposures.^42^ Congenital cardiovascular abnormalities have been associated with maternal HIV infection, maternal diabetes, maternal febrile illness, certain drugs and chemicals, and deficiencies in particular nutrients during gestation. In addition, low birth weight for gestational age has been associated with adult health outcomes including acute myocardial infarction.^43^ The impact of prenatal exposure to maternal influenza infection on offspring has been evaluated by others. There is evidence from rodent studies, suggesting that prenatal exposure to cytokines, specifically IL-6 which is produced in response to influenza and other inflammatory processes, is associated with hypertension in adult offspring.^44^,^45^ As discussed earlier, the relationship between *in utero* exposure to the 1957 influenza pandemic and development of schizophrenia has been studied,^17^–^22^ and several groups have examined birth cohort effects associated with the 1918 pandemic.^23^–^25^

Mazumder et al. reported an increase in cardiovascular disease among Americans born in 1919 compared with those born in the years immediately before (1915–1918) and after (1920–1923).^25^ While that study examined self-reported prevalent cardiovascular disease among persons aged 60–82 years, our study examined registry-recorded events that occurred during ages 62–87 for the 1918 pandemic-related cohorts. These differences in study design could partly explain the difference in results.

In conclusion, we did not observe an association between prenatal influenza exposure and either acute MI or stroke in the present study. The results are generally consistent across the three pandemics. The pandemics allowed us to consider the association of interest with varying age ranges for incident events as well as with different influenza strains (H1N1 in 1918, H2N2 in 1957, and H3N2 in 1968), adding strength to our finding of a uniformly null association. We have performed a fairly comprehensive evaluation of the associations of interest over different age ranges, although survivor bias and left truncation of the outcomes for the 1918 pandemic may have impacted our findings. The current relatively young ages of the persons included in the 1957 and 1968 analyses may warrant re-evaluation at a later time.
